# Promoting walking among office employees ― evaluation of a randomized controlled intervention with pedometers and e-mail messages

**DOI:** 10.1186/1471-2458-12-403

**Published:** 2012-06-06

**Authors:** Minna Aittasalo, Marjo Rinne, Matti Pasanen, Katriina Kukkonen-Harjula, Tommi Vasankari

**Affiliations:** 1 The UKK Institute for Health Promotion Research, P.O. Box 30, FI-33501, Tampere, Finland; 2 National Institute for Health and Welfare, P.O. Box 30, FI-00271, Helsinki, Finland

**Keywords:** Physical activity, Health promotion, pedometer, intervention, worksite, evaluation, RE-AIM

## Abstract

**Background:**

The purpose of the study was to evaluate a 6-month intervention to promote office-employees’ walking with pedometers and e-mail messages.

**Methods:**

Participants were recruited by 10 occupational health care units (OHC) from 20 worksites with 2,230 employees. Voluntary and insufficiently physically active employees (N = 241) were randomized to a pedometer (STEP, N = 123) and a comparison group (COMP, N = 118). STEP included one group meeting, log-monitored pedometer-use and six e-mail messages from OHC. COMP participated in data collection. Reach, effectiveness, adoption, implementation, maintenance (RE-AIM) and costs were assessed with questionnaires (0, 2, 6, 12 months), process evaluation and interviews (12 months).

**Results:**

The intervention *reached* 29% (N = 646) of employees in terms of participation willingness. Logistic regression showed that the proportion of walkers tended to increase more in STEP than in COMP at 2 months in “walking for transportation” (Odds ratio 2.12, 95%CI 0.94 to 4.81) and at 6 months in “walking for leisure” (1.86, 95%CI 0.94 to 3.69). Linear model revealed a modest increase in the mean duration of “walking stairs” at 2 and 6 months (Geometric mean ratio 1.26, 95%CI 0.98 to 1.61; 1.27, 0.98 to 1.64). *Adoption* and *implementation* succeeded as intended. At 12 months, some traces of the intervention were sustained in 15 worksites, and a slightly higher number of walkers in STEP in comparison with COMP was observed in “walking stairs” (OR 2.24, 95%CI 0.94 to 5.31) and in “walking for leisure” (2.07, 95%CI 0.99 to 4.34). The direct *costs* of the intervention were 43 Euros per participant.

**Conclusions:**

The findings indicate only modest impact on some indicators of walking. Future studies should invest in reaching the employees, minimizing attrition rate and using objective walking assessment.

**Trial registeration:**

ISRCTN79432107

## Background

The benefits of physical activity (PA) in the prevention, treatment and rehabilitation of major chronic diseases are well recognized. According to the most recent recommendations, to promote and maintain cardiovascular fitness and health, all adults should accumulate a minimum of 150 weekly minutes of moderate-intensity aerobic PA spread throughout the week [1]. Based on the most recent survey in Finland, only half of the adult population meets this recommendation [2]. At worksites inactivity can lead to increased sick leaves, impaired workability and ultimately higher productivity costs [3].

Employers’ traditional efforts to promote PA, e.g. incentives to participate in structured exercise or events, usually attract only 30% or even fewer of the employees [4]. Furthermore, the majority of those participating are already physically active and healthy [5]. Actions aiming at promoting lifestyle activities, such as walking, which can be adapted to everyday life more easily, are therefore needed. Walking is the most popular PA mode and accessible to most people regardless of age, sex, income level or skills. Moreover, walking has significant potential in reducing the incidence of chronic diseases and promoting health-related equality with the minimum number of adverse effects [6].

In recent years, pedometers have been used widely in campaigns at national, community and worksite level to promote walking (e.g. http://www.coloradoonthemove.orghttp://www.canadaonthemove.cahttp://www.10000steps.org.au). Pedometers are simple to use, low-cost, accurate [7] and demand less staff resources than traditional face-to-face approaches. They also give immediate and explicit feedback to the users. Together with self-evaluation and self-reinforcement, self-monitoring may help individuals to develop self-regulatory skills for behavior change [8] and be a powerful behavior change technique [9].

Overviews and studies on pedometer-based interventions conclude that self-monitoring may have positive short-term effects on PA in general population [10-13]. However, the most recent systematic review [10] identified only five worksite interventions none being a randomized controlled trial (RCT). Two of the interventions examined pedometer-use only [14,15], one intervention added a weekly meeting to facilitate pedometer-use [16] and two interventions supported pedometers with weekly e-mails [17,18].

The results from pedometer-only studies were contradictory: one [14] found no motivational effect whereas the other [15] discovered a significant increase in the number of daily steps. The findings from the multifaceted interventions were more consistent and in all of them an increase in the daily steps was observed [16-18]. The results of two non-RCT’s [19,20] and one RCT [21] published after the review and using pedometers and e-mail messages are encouraging as well.

The purpose of this study was to evaluate a 6-month pedometer-based intervention supported with a monthly e-mail message in an office-based worksite setting by using a randomized controlled design. The evaluation followed the principles of RE-AIM [22] including five dimensions: reach, effectiveness, adoption, implementation and maintenance. In addition, the direct costs of the intervention were assessed. It was hypothesized that the intervention enhances different types of walking and decreases sedentary time during working and non-working day and is feasible and low-cost.

## Methods

### Sample and instrumentation

The head of occupational health care services in 13 occupational health care units (OHC) in Southern Finland were contacted by e-mail to participate in the study. Ten of them volunteered and recruited 20 office-based worksites to the study involving altogether 2,230 employees. The study was conducted according to the guidelines of good scientific practice by The National Advisory Board on Research Ethics in Finland (http://pro.tsv.fi/tenk/ENG/Function/htkeng.pdf) and approved by the independent ethical committee of the UKK Institute.

An employer-representative named in each worksite delivered the baseline questionnaires to the employees, who completed and returned them by mail to the research institute. Background questions included year of birth, gender, marital status, care-giver to children under 18 years of age (yes/no), education, occupation, number of weekly working hours, physical loading of the work, subjective estimation of present work ability compared with the lifetime best (scale 0–10) [23], self-reported health status (good, fairly good, average, fairly poor, poor), restrictions for PA (no, some, severe, prevents totally), height and weight. Primary outcome questions were “walking at work”, “walking for transportation”, “walking for leisure” and “walking stairs” as well as sedentary time during working and non-working day. Vigorous and moderate-intensity leisure PA other than walking was also elicited, but are not reported here since increasing walking was the primary target of the intervention.

Questions on walking and sitting were adopted from the self-administered long version of International Physical Activity Questionnaire for a usual week (IPAQ; http://www.ipaq.ki.se) [24]. However, the questions on PA at work and during transportation deviated from the original ones since only walking was elicited. Also, a question on walking stairs, which was specifically promoted in the intervention, was added by using the same form as in other walking questions. Follow-up questionnaires at 2, 6 and 12 months after the preliminary meeting were mailed to participants’ home addresses based on the information they had given in the baseline questionnaire. The baseline and follow-up questionnaires were used as outcome measures of this study.

Respondents to the baseline questionnaire were eligible to participate if they volunteered for the study and were insufficiently physically active for cardiorespiratory health (= less than 150 minutes of moderate-intensity PA or less than 75 minutes of vigorous-intensity PA per week accumulated from fewer than 3 days a week) [1] and perceived no restrictions for PA. Eligible respondents at each worksite were then allocated equally either to a pedometer (STEP) or a comparison group (COMP) using computer-generated randomization lists (stratified randomization with random allocation sequences to ensure closely balanced groups within each worksite).

### Intervention

### The employees in STEP participated in a 6-month intervention consisting of

1) A 1-hour preliminary meeting in each worksite held by a researcher and providing information on the intervention as well as on health benefits and recommendations of PA and walking. The use of stairs was emphasized from the aspect of health and easy applicability. The employees were also supplied with walking leaflets, pedometers (Omron, Walking Style II) and printed logbooks

2) Self-monitoring of PA with the pedometer and logbook

3) Monthly e-mail message from OHC.

In the preliminary meeting the employees in STEP were instructed to assess their average number of daily steps with a pedometer during three days including two working and one non-working day, which has been shown to be sufficient for predicting weekly PA ([25]). The average number of daily steps was used as the baseline for the further step goals, which were prompted monthly by the logbooks and e-mail messages sent by the OHC. More frequent e-mail messaging has been used in previous studies [19,20], but a monthly approach was chosen for this study because the duration of the intervention was considerably longer and to ensure the transferability of the intervention to the routine OHC practices. Tailoring the step goals according to the individual’s baseline activity was chosen rather than using general goals such as taking 10,000 steps per day [26].

The Health Action Process Approach (HAPA) [27-29] was used as a context in formulating the e-mail messages (Table 1). The ultimate goal was to add 4000 steps approximating 30 minutes of moderate-intensity walking [30] to the daily baseline on five self-selected days of the week. The goal was approached progressively until the 5^th^ e-mail by setting smaller goals of increase in a sequence of 2000 steps, which has been shown achievable at worksite setting [15].

**Table 1:** **Contents of e-mail messages using the Health Action Process Approach (HAPA) model as the framework**[27-29]

	**Timing**	**Elements of HAPA**	**Content of the e-mail message**
**ORIENTATION**			
Preliminary meeting		Risk perception	· Information on the intervention, benefits of physical activity (PA), PA recommendations and walking
			· Instructions on monitoring PA with a pedometer and a logbook and assessing the average number of daily steps at baseline
			· Presentation of the ultimate goal of adding 4000 moderate-intensity steps to the baseline on 5 days of the week
**MOTIVATIONAL PHASE (INTENTION BUILDING)**			
1^st^ e-mail *“Regaining strength by physical activity”*	Within 2 weeks from the preliminary meeting	Positive outcome expectations, pre-action self-efficacy, action planning	· Benefits of integrating short bouts of PA into daily routine
			· The 1^st^ goal of adding 2000 steps to the baseline on 2 days of the week
			· Simple tips for accumulating 2000 steps
**VOLITIONAL PHASE (INTENTION): PLANNING, PREPARING AND INITIATING PHYSICAL ACTIVITY**			
2^nd^ e-mail *“Finding time through experience”*	4 weeks after the 1^st^ e-mail	Positive outcome expectations, risk perception, action planning, coping planning, self-monitoring	· Positive outcomes after even a short bout of PA
			· Examples of finding time and places to be more active
			· The 2^nd^ goal of adding 2000 steps to the baseline on 5 days of the week
3^rd^ e-mail *“Making physical activity one’s own thing”*	4 weeks after the 2^nd^ e-mail	Positive outcome expectancies, risk perception, coping planning, maintenance self-efficacy, action planning	· Positive outcomes from being physically active
			· Examples of accumulating 4000 steps
			· The 3^rd^ goal of adding 4000 steps on 2 days and 2000 steps on 3 days of the week to the baseline
**VOLITIONAL PHASE (MAINTENANCE)**			
4^th^ e-mail *“Preparing oneself against barriers”*	4 weeks after the 3^rd^ e-mail	Action planning, coping planning, maintenance planning, maintenance self-efficacy	· The importance of regularity in PA
			· The most critical barriers for PA and the ways to overcome them
			· Example of a 30-minute walking session with 4000 steps
			· The 4^th^ goal of adding 4000 steps on 4 days and 2000 steps on 1 day of the week to the baseline
5^th^ e-mail *“Getting oneself going”*	4 weeks after the 4^th^ e-mail	Action planning, coping planning, maintenance self-efficacy, recovery self-efficacy	· Tips for making it easier to “get oneself going”
			· The 5^th^ goal of adding 4000 steps to the baseline on 5 days of the week
6^th^ e-mail *“Establishing a physically active lifestyle”*	4 weeks after the 5^th^ e-mail	Action self-efficacy, maintenance self-efficacy, recovery self-efficacy, action planning	· Learning sustainable ways to be physically active
			· The importance of regularity and the possibility of rewarding oneself
			· Maintenance of current PA level and supplementation with muscle strengthening exercises on 2 days of the week
			· A printable form for a weekly action plan and monitoring

In the first e-mail, for example, the employees were encouraged to add 2000 steps to their baseline on 2 days of the week. They were advised to choose the particular days from the following week and to enter the step goal (baseline + 2000) to the logbook on the day-specific space. For the remaining 5 days, the baseline step count was entered as the goal. The employees also made daily action plans in the logbook on how they were going to reach the step-goals, as suggested by Schwarzer et al. [29]. In the end of each day they entered the steps taken to the logbook and assessed how they had met the daily goal.

In COMP only data collection took place. However, after the 12-month follow-up a 1-hour meeting was offered to each worksite to provide feedback to all the employees and to supply the participants in COMP with pedometers, logbooks and walking leaflets. In addition, as a gesture of appreciation each participating OHC was handed out 10 point-of-choice stair posters and offered a 2-hour training session on PA and health for the health personnel.

### Evaluation

The evaluation was based on the RE-AIM framework [22] including five dimensions: reach, effectiveness, adoption, implementation and maintenance (http://www.re-aim.org). The framework is recommended for the evaluation of health promotion interventions for more systematic balancing of internal and external validity [e.g. [31,32], which is needed for translating the study results into practice. Evaluation questions, indicators and measures of the present intervention are described in Table 2. As seen from the effectiveness, the pedometers were used as motivators, not as outcome measures.

**Table 2:** **Evaluation of the intervention based on the RE-AIM framework**[22]

**Dimension**	**Evaluation question**	**Indicator**	**Measure**
Reach	to what extent were the occupational health care units (OHC) and employees willing to take part and how representative were they?	Number of OHC’s and employees responding and being willing to participate in the study	Process evaluation and baseline questionnaire
Effectiveness	What impact did the intervention have on participants walking and sitting?	Weekly minutes of walking at work, for transportation, in stairs, for leisure and of total walking; Daily minutes of sitting during a working and a non-working day	Baseline questionnaire and follow-up questionnaires at 2 and 6 months
	Did the intervention cause negative outcomes (adverse effects)?	Incidence of adverse effects due to physical activity	Follow-up questionnaires at 2 and 6 months
Adoption	To what extent did the recruited agents and participants complete the study?	Number of occupational health care units, worksites and participants completing the study.	Process evaluation and follow-up questionnaires at 2 and 6 months
Implementation	Were the various intervention actions delivered as intended?	Setting level: Delivery of e-mail messages Individual level: Attendance to the preliminary meeting; Use of pedometers and logbooks, receiving and reading e-mail messages	Notes kept by the researcher; follow-up questionnaires at 2 and 6 months; standardized records kept by the occupational health care
Maintenance	To what extent were the intervention actions maintained?	Setting level: The number of worksites where some of the intervention actions existed 6 months after the cessation of the study	Telephone-interview of the employer-representatives at 12 months
	What were the long-term effects?	Individual level: Changes in walking, sitting and subjective work ability 6 months after the cessation of the study	Baseline questionnaire and follow-up questionnaire at 12 months

Direct costs of the intervention were assessed by accumulating the costs related to 1) the working time spent by the employer-representatives for the study arrangements (semi-structured telephone-interview at 12-months by a research assistant), 2) the working time spent by the OHC personnel for delivering each e-mail message (OHC-specific structured form), 3) the working time spent by the researcher for the preliminary meetings and 4) the prices of pedometers, logbooks and walking leaflets (researchers’ notes).

### Statistics

The required sample size for the change in the weekly minutes of total walking was estimated by utilizing Finnish data from a previous IPAQ study [24]. It was hypothesized that the weekly minutes will increase 30% more in STEP than in COMP. The duration at baseline was estimated to be 150 minutes per week in both groups. According to the power calculations (significance level of 0.05, power of 80%) 175 participants in each group totaling 350 participants were needed to detect the 30% between-group difference in change in the weekly minutes of total walking.

Self-reported data on different types of walking were analyzed by using Generalized Linear Mixed Models (GLMM). Due to the excess number of zeros (no walking) in the data the analysis was performed in two parts as suggested by Tooze et al. [33]:

1) Logistic regression to analyze the between-group difference in change in the probability (odds ratio, OR) of walking from the baseline to the 2, 6 and 12-month follow-ups. The data were coded dichotomously into zeros (0 = no walking) and nonzeros (1 = walking).

2) Linear model to estimate the between-group changes in nonzero responses. In this model, the measurements indicated repetitions with the assumption that the correlations between the repeated measurements were constant (compound symmetry). As most of the distributions were skewed logarithm transformation were performed and geometric mean ratio (GMR) was used as an indicator of group differences. Worksite was included in the model as a random effect (intercept) and gender, taking care of children less than 18 years of age (yes/no), age (continuous) and self-reported body mass index at baseline (continuous) were included as covariates. The random errors were assumed to be independent between the two model components. This type of analysis for repeated measures allows incorporation of incomplete longitudinal data into the models.

Linear model only with the same covariates as in the different types of walking was used to analyze the between-group difference in change in total walking (GMR) and sitting at 2, 6 and 12-month follow-up and in subjective work ability at 12-month follow-up. Fisher’s exact test was performed to analyze the group difference in the incidence of adverse events due to PA at 2 and 6-month follow-up.

## Results

### Reach

Ten OHC’s participated in the study. The reach in relation to OHC was therefore 77%. All the participating OHC’s supplied services to several companies representing the model, which is the most common in Finland (Statistical Yearbook of the Social Insurance Institution 2010, http://www.kela.fi/it/kelasto/kelasto.nsf/alias/Yearbook_10_pdf/$File/Yearbook_10.pdf?OpenElement). Of the OHC’s not participating in the study, two provided services only to their own company and one was equal to the participating OHC’s.

**Figure 1:**
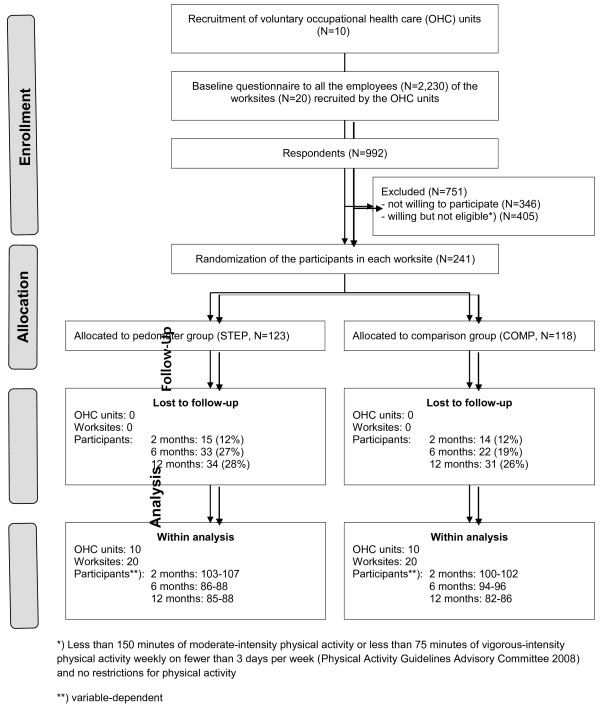
Flow-chart of the study.

The response rate to the baseline questionnaire in the worksites varied from 32% to 72% (see Additional file 1). Of respondents, 646 (65%) were willing to participate and 241 (24%) met the further eligibility criteria of being insufficiently physically active for cardiovascular health and perceiving no restrictions for PA (Figure 1). The percentage of employees willing to participate ranged from 52% to 85% and the proportion of insufficiently physically active employees from 7% to 60% in different worksites. There seemed to be no systematic tendency in the response rate, willingness rate or eligibility in terms of the size of the worksite. Thus, the reach of the intervention was 29% in terms of employees’ willingness to participate in the intervention.

### Effectiveness

Of the 241 participants 123 were randomized to STEP and 118 to COMP (Figure 1). Compared to COMP the participants in STEP were more often women, less frequently taking care of minors and less often overweight (Table 3). Participants’ walking and sitting at baseline are shown in Table 4.

**Table 3:** Baseline characteristics of the participants in the pedometer (STEP) and comparison group (COMP)

	**Participants**	
	**STEP (N = 123)**	**COMP (N = 118)**
Age (years), mean (SD)	44.1 (9.4)	45.3 (9.1)
Women, N (%)	87 (71)	78 (66)
Married, N (%)	99 (81)	96 (81)
Taking care of children under 18 years of age, N (%)	61 (50)	69 (59)
Education, N (%)		
Basic	7 (6)	11 (9)
Polytechnic or vocational school	79 (64)	75 (64)
University degree	37 (30)	32 (27)
Working hours per week, mean (SD)	37.4 (6.1)	37.1 (6.8)
Perceived physical loading at work, N (%)		
Sedentary	113 (93)	110 (93)
Mainly standing or light mobility	8 (6)	8 (7)
Heavy	1 (1)	0 (0)
Subjective work ability (scale 0–10), mean (SD)	8.0 (1.3)	8.0 (1.4)
Perceived health, N (%)		
Good or fairly good	82 (67)	76 (64)
Average	35 (29)	34 (29)
Fairly poor or poor	5 (4)	8 (7)
Body mass index > 25 kg/m^2^, N (%)	63 (51)	76 (64)

Means or proportions. N = number of participants.

**Table 4:** Weekly minutes of walking, proportion of walkers and daily minutes of sitting at baseline and at 2, 6 and 12-month follow-up in the pedometer (STEP) and in the comparison (COMP) group

	**Baseline**		**2 months**		**6 months**		**12 months**	
	**STEP**	**COMP**	**STEP**	**COMP**	**STEP**	**COMP**	**STEP**	**COMP**
**Walking at work, N**	**121**	**117**	**103**	**102**	**87**	**95**	**88**	**82**
·Weekly minutes, mean (SD)	144 (209)	157 (236)	150 (159)	174 (239)	158 (186)	161 (254)	172 (191)	145 (155)
·Walkers, %	98	96	98	98	99	94	99	96
**Walking for transportation, N**	**121**	**115**	**106**	**102**	**87**	**96**	**85**	**85**
·Weekly minutes, mean (SD)	115 (172)	134 (168)	134 (142)	163 (203)	136 (147)	138 (172)	170 (298)	127 (172)
·Walkers, %	77	81	88	79	88	86	83	79
**Walking stairs, N**	**120**	**117**	**105**	**100**	**86**	**96**	**87**	**87**
·Weekly minutes, mean (SD)	38 (45)	46 (100)	62 (65)	49 (60)	67 (89)	46 (52)	63 (63)	56 (79)
·Walkers, %	86	81	91	79	91	86	93	79
**Walking for leisure, N**	**122**	**117**	**106**	**100**	**88**	**95**	**88**	**86**
·Weekly minutes, mean (SD)	72 (96)	68 (125)	105 (96)	90 (119)	96 (90)	80 (133)	115 (130)	79 (102)
·Walkers, %	74	71	87	84	85	70	87	76
**Total walking, N**	**117**	**112**	**101**	**99**	**86**	**94**	**84**	**80**
·Weekly minutes, mean (SD)	370 (311)	400 (401)	455 (255)	465 (433)	457 (306)	431 (403)	521 (468)	395 (319)
·Walkers, %	100	100	100	100	100	99	99	99
**Sitting during a working day, N**	**121**	**118**	**107**	**102**	**87**	**94**	**87**	**85**
·Daily minutes, mean (SD)	544 (152)	554 (161)	502 (147)	513 (175)	503 (165)	520 (169)	477 (159)	510 (183)
**Sitting during a non-working day, N**	**121**	**117**	**105**	**100**	**87**	**96**	**87**	**87**
·Daily minutes, mean (SD)	347 (165)	382 (180)	333 (168)	339 (170)	331(156)	363 (176)	300 (153)	347 (165)

Minutes are shown as arithmetic means and standard deviations (SD).

Between-group differences in changes in walking and sitting are presented in Table 5. In *“walking for transportation”* the logistic regression analysis showed that at 2 months slightly more participants started walking in STEP than in COMP (14 vs. 6 employees) as opposed to those who stopped walking (7 vs. 6 employees) (OR 2.12, 95%CI 0.94 to 4.81). This tendency was no longer visible at 6-month follow-up. In the weekly minutes no between-group difference in change was discovered at 2 AND 6-month follow-up (GMR 0.84, 95%CI 0.64 to 1.11; GMR 1.08, 95%CI 0.82 to 1.44). In *“walking for leisure”*, similarly, although not until at 6 months, the number of new walkers (7 vs. 11 employees) against to those who stopped walking (0 vs. 11), was somewhat greater in STEP than in COMP (OR 1.86, 95%CI 0.94 to 3.69). Again, no between-group difference in change was apparent in the weekly minutes at either of the follow-up (GMR 1.22, 95%CI 0.96 to 1.54; GMR 1.09, 95%CI 0.85 to 1.39). In *“walking stairs”* the logistic regression revealed no difference in change between the groups in the proportion of new walkers at 2 or 6-month follow-up (OR 1.44, 95%CI 0.64 to 3.25; OR 0.97, 95%CI 0.40 to 2.34) but the linear model showed a modest between-group difference in change in the weekly minutes at 2 and 6 months (GMR 1.26, 95%CI 0.98 to 1.61; GMR 1.27, 95%CI 0.98 to 1.64). No difference in change between the groups was discovered at 2 and 6 months in *sitting during a working day* (−3 minutes/day, 95%CI −45 to 40;-4 minutes/day, 95%CI −46 to 38), and during *a non-working day* (30 minutes/day, 95%CI −9 to 70; 1 minute/day, 95%CI −42 to 44) and in the *incidence of adverse effects* due to PA (8% vs. 14% and 15% vs. 17%).

**Table 5:** Difference in change between the pedometer (STEP) and comparison group (COMP) in the weekly minutes of walking and in the daily minutes of sitting at 2, 6 and 12-month follow-up and in subjective work ability at 12-month follow-up

	**Logistic regression**^**a**^			**Linear model**^**b**^		
	**Odds ratio 95% confidence interval**			**Geometric mean ratio 95% confidence interval**		
	**2 months**	**6 months**	**12 months**	**2 months**	**6 months**	**12 months**
Walking at work	0.73 0.06 to 8.89	4.20 0.31 to 57	2.39 0.15 to 37.3	1.11 0.92 to 1.35	1.09 0.89 to 1.32	1.13 0.92 to 1.38
Walking for transportation	2.12 0.94 to 4.81	1.28 0.53 to 3.12	1.57 0.68 to 3.61	0.84 0.64 to 1.11	1.08 0.82 to 1.44	1.03 0.77 to 1.39
Walking stairs	1.44 0.64 to 3.25	0.97 0.40 to 2.34	2.24 0.94 to 5.31	1.26 0.98 to 1.61	1.27 0.98 to 1.64	1.18 0.91 to 1.53
Walking for leisure	1.18 0.58 to 2.40	1.86 0.94 to 3.69	2.07 0.99 to 4.34	1.22 0.96 to 1.54	1.09 0.85 to 1.39	1.21 0.94 to 1.55
Total walking	**·**	**·**	**·**	1.19 0.95 to 1.49	1.19 0.95 to 1.51	1.25 0.98 to 1.59
				Mean difference^c^ 95% confidence interval		
				2 months	6 months	12 months
Sitting during working day	**·**	**·**	**·**	−3 −45 to 40	−4 −46 to 38	−9 −56 to 37
Sitting during non-working day	**·**	**·**	**·**	30 −9 to 70	1 −42 to 44	−9 −52 to 33
Subjective work ability(scale 0–10)	**··**	**··**	**··**	**··**	**··**	0.3 −0.1 to 0.6

^a^ The probability (odds ratio) of walking from baseline to 2, 6 and 12-month follow-up. The data were coded dichotomously to zeros (=0, no walking) and nonzeros (=1, walking).

^b^ Logarithm-transformed nonzero responses. Worksite included as a random effect (intercept) and gender, taking care of minors (yes/no), age (continuous) and body mass index at baseline (continuous) as covariates.

^c^ Untransformed values.

Category not applicable.

Data not available.

### Adoption

From the participating 10 OHC and 20 worksites all carried out the intervention representing 100% of the initial sample of OHC and worksites. The number of participants lost in follow-ups was 29 (12%) at 2 months, 55 (23%) at 6 months and 65 (27%) at 12 months. The attrition rate at 6 months was greater in STEP (27%) than in COMP (19%).

### Implementation

At the setting level, the messages were delivered as intended in all but one OHC, where the 6^th^ e-mail message was one month late. At the individual level, eighteen (15%, range 0% to 60% in individual worksites) employees in STEP did not attend the preliminary meeting. At the 12-month follow-up, 60% of the participants in STEP reported having used pedometers regularly and 37% irregularly during the 6-month intervention. The corresponding percentages in the logbooks were 46 and 47. The e-mails reached 98% of the participants at 2 months and 99% at 6 months. As recalled by the participants, the mean number of messages received was 2 (SD 0.7) at 2 months and 5 (1.1) at 6 months. At 6 months, 80% of the participants reported they had read the messages.

### Maintenance

At the setting level, based on the interviews of the employer-representatives, actions to promote PA during the 6 months after the cessation of the intervention had been taken at nine (45%) worksites. Indications of some traces of the intervention were seen in 6 (30%) worksites: in 2 worksites many of the employees were still using the pedometer, in 2 worksites the OHC had started to promote PA with pedometers, in 1 worksite stair-use had been emphasized more intensively and in 1 worksite a competition about the number of steps between the administration and other employees had been initiated.

At the individual level, the logistic regression revealed a slim between-group difference in change at 12 months in *“walking stairs”* (OR 2.24, 95%CI 0.94 to 5.31) and in *“walking for leisure”* (OR 2.07, 95%CI 0.99 to 4.34) in favor of STEP (Table 5). No difference in the change was discovered between the groups in work ability (0.3 points in the scale of 0–10, 95%CI −0.1 to 0.6).

### Costs

Thirteen (62%) of the 20 employer-representatives were reached for the telephone interview at 12-months. Their estimations about the time spent for the study varied from ½ to 5 hours with the average of 2.6 hours. Four of the representatives interviewed were unable to estimate the accumulative costs. The other nine reported costs varying from 20 to 300 Euros with the average of 128 Euros equaling 2560 Euros for all the worksites. Each OHC spent on average 28 minutes (range 2 to 90 minutes for all the messages and 36 seconds to 15 minutes for one message) for sending the 6 e-mail messages to the participants of single worksite. With the average monthly salary of the health care personnel in the private sector in 2009 (Statistics Finland, http://www.stat.fi/til/pra/2009/pra_2009_2011-04-08_tau_001_en.html) the average cost per worksite was 8 Euros [monthly salary of 2535 Euros/(21x7.5 h) = hourly salary of 16 Euros] equaling 160 Euros for all the worksites. Costs related to the researchers’ working time for the meetings, pedometers, printed logbooks and walking leaflets were altogether 2617 Euros. As a result, the direct costs of the intervention were 5337 Euros, which makes approximately 43 Euros per participant in STEP.

## Discussion

The purpose of the study was to RE-AIM evaluate a minimal intervention to promote walking and reduce sitting among office employees in 20 worksites. The 6-month intervention consisted of a preliminary group meeting, self-monitoring of PA with a pedometer, printed logbook and a monthly e-mail message from OHC. The intervention *reach* was 77% in OHC’s and 29% in employees. Only modest effects were discovered in “walking stairs” and “walking for transportation” at 2 months and in “walking for leisure” and “walking stairs” at 6 months. The intervention was safe causing no excessive number of adverse effects. All the companies and OHC recruited *adopted* the intervention but at the individual level the drop out rates at follow-ups somewhat deteriorated adoption. *Implementation* was carried out as intended and at the setting level some of the actions were *maintained* until 6 months after the intervention in three fourths of the companies. At the individual level the small between-group difference in change in favor of STEP was still apparent in “walking stairs” and “walking for leisure” at 12-months. The intervention was considerably low-cost.

### Reach

The high reach of OHC’s indicated their interest in obtaining simples strategies to promote PA at worksites. The OHCs participating were also representative in terms of providing their services. The relatively high number of participating companies improved the generalizability of the study results. At the same time it is, nevertheless, recognized that the large proportion of non-respondents in employees caused selectivity to the sample. Based on earlier studies it is probable that a notable part of insufficiently physically active employees resigned from the study [34,35] and primarily the most compliant ones participated.

### Effectiveness

The intervention was able to affect only modestly some of the outcomes of walking. The scarce findings may partly be explained by the outcome measure: Self-reports are known to produce greater variation in PA-related estimates than more objective measures such as accelerometers or pedometers and to have inadequate sensitivity for detecting intervention effects [36,37]. However, in this study, pedometers were used as motivators, not as outcome measures. To utilize pedometers for outcome purposes, COMP should have been provided not only with the pedometers but also with the logbooks, which would have exposed COMP to a minimal intervention and diminished the difference between the intervention arms. As a result, a larger sample size may have been needed to discover the changes in walking. Sealing of the pedometers for the period of data collection would not have been possible either because it would have interrupted the log-monitoring in STEP, which was the main element of the intervention. On these grounds the most applicable method for the more objective data collection would have been the concurrent use of accelerometers.

It should, furthermore, be noted that the conditions according to the power calculations were not fully accomplished impairing the detection of group differences. It is also possible that the individual-level randomization within each worksite caused contamination and diluted the difference in change between the intervention arms. However, as seen in Table 4, no favorable changes occurred in COMP indicating low level of contamination. This may be explained by the fact that the participants in STEP were urged in the preliminary meeting not to discuss about the intervention with their colleagues. They were also informed that the participants in COMP would receive the material after the intervention. Nevertheless, cluster randomization may be a better alternative in future studies to minimize contamination. Then a larger sample size in total and in each cluster would be needed to adjust the statistical analysis to intra-cluster variation.

It is also possible that a single face-to-face contact and an e-mail message once a month was not sufficient to comply with the pedometer-based goals. This may particularly apply to physically inactive employees, who may need more external support for behavioral change. On the other hand, most of the modest between-group differences in changes were discovered in the proportions of participants starting to walk as opposed to those terminating walking suggesting that the intervention was able to engage employees with low walking activity. Considering the high number of walkers at baseline in all types of walking substantially greater changes may be expected in a more inactive population [21]. Regardless of the slightly larger increase in the number of non-walkers, STEP had no more adverse effects than COMP, which is in line with studies on injury rates of various PA modes [38] and is an important aspect also from the employers’ point of view.

The intervention did not seem to have any effect on “walking at work”. This is not unexpected because more intensive actions than included in this study seem to be needed to promote short PA breaks during working hours [39]. What was more distracting was that although the intervention had some impact on walking during leisure time it did not carry over to time spent in sitting during a working or a non-working day. This is, however, explainable through recent studies, which indicate that both overall sitting [40] and occupational sitting [41] are independent risk factors for health and not necessarily associated with the amount of PA [21,42,43]. Also, the effects on walking may have been too small to show changes in sitting.

### Adoption

All the companies and OHC completed the study. This reflects that the presumptions about the abilities of the companies and OHC to carry out the study were appropriate. At the individual level the drop out rate especially at 6-months deteriorated adoption. The drop out rate was equal to the study of Dinger et al. [19] and substantially lower than in the non-controlled study of Faghri et al. [20] both representing similar although much shorter (6 and 10 weeks) interventions in the worksite setting compared the present study. Pedometer studies supported with weekly personal contacts do not necessarily attain lower attrition rates as shown in the 12-week non-controlled study of Chan et al. [16], where 25% of the sedentary workers dropped out before eight weeks’ data collection.

### Implementation

The participation of employees in the preliminary meeting was good. The employees were informed about the meeting several days before via e-mail. The employer-representative, who was present in the meeting at each worksite, was responsible after the meeting for providing the information, material and pedometers for those not attending. The use of pedometers and logbooks was also satisfactory although the proportion of regular users could have been better especially for logbooks (46%). The number of e-mail messages recalled by the participants as having received was in accordance with the actual number of messages delivered and the majority of the participants reported reading them. Due to successful implementation it is probable that the modest effects on walking resulted from the type of intervention intended.

### Maintenance

*At the setting level* the findings on maintenance are encouraging since at 12 months some traces of the intervention still existed in three fourths of the companies without targeting extra efforts on dissemination. This may reflect the adoptability of the intervention due to its simplicity. It seemed that the intervention was able to achieve some sustainability *at the individual level* in “walking for leisure”. Most of the pedometer-based studies have been of short duration [44] implying that there is a lack of comparable research about sustainability. No impact on work ability was detected, which is not surprising because of the complexity of work ability as well as of the fact that the intervention was light and did not target at any other mediators of work ability than PA.

### Costs

The direct costs of the intervention were low indicating good transferability of the intervention to the practice.

## Conclusions

The study evaluated the reach, effectiveness, adoption, implementation and maintenance (RE-AIM) as well as direct costs of a simple pedometer-based intervention in 20 Finnish worksites in a randomized setting. The reach of the intervention was acceptable. Only modest short- and long-term impact was detected in “walking stairs”, “walking for transportation” and “walking for leisure”. The intervention seemed safe, inexpensive and highly adoptable in worksite setting.

The findings indicate only small changes in some indicators of walking with a simple and low-cost intervention using pedometers and e-mail messages. More intensive approach including multiple face-to-face contacts and/or more frequent e-mail messaging particularly in the beginning of the intervention may possibly be needed to achieve significant impact on walking. However, this intervention was operated under real-world conditions and, except for the preliminary meeting, conducted by using the existing structures and resources of the worksites and their OHC. This improves the transferability and applicability of the results to practice.

In the future studies more effort should be put on reaching as many of the employees as possible for eligibility assessment. This would help in estimating the selectivity of the sample and moreover the generalizability of the findings. Minimizing the attrition rate improves the representativeness of the results. Further, concurrent use of accelerometers or other blinded objective methods to assess walking and adequately powered sample size would be needed to confirm the findings on effectiveness. Finally, multilevel analysis with a larger sample size in each worksite would provide important information about the impact of type of worksite and would thus better enable tailoring of interventions. A larger sample size obtained from a certain population may also allow the use of WHO’s Health Economic Assessment Tool (HEAT, http://www.euro.who.int/HEAT) and the estimation of economic savings as a consequence of increased walking.

## Competing interests

The authors declare that they have no competing interests.

## Authors’ contributions

MA conceived the study, coordinated the study together with MR and is the principal author. MR, MP, KKH and TV participated in designing the study and in drafting the manuscript. MP performed the statistical analysis. All authors read and approved the final manuscript.

## Pre-publication history

The pre-publication history for this paper can be accessed here:

http://www.biomedcentral.com/1471-2458/12/403/prepub

## Supplementary Material

Additional file 1 Number (N) of employees, respondents, respondents willing to participate, and respondents willing to participate and meeting the inclusion criteria of being insufficiently physically active and perceiving no restrictions for physical activity. Word-document. Click here for file
