# Supplementary material for: Promoting walking among office employees ― evaluation of a randomized controlled intervention with pedometers and e-mail messages
Source: BMC Public Health. 2012 Jun 6;12:403. doi: 10.1186/1471-2458-12-403 (PMC3444317; doi:10.1186/1471-2458-12-403)
Supplement: Additional file 1 — Number (N) of employees, respondents, respondents willing to participate, and respondents willing to participate and meeting the inclusion criteria of being insufficiently physically active and perceiving no restrictions for physical activity. Word-document. [file 1471-2458-12-403-S1.docx]

Appendix. Number (N) of employees, respondents, respondents willing to participate, and respondents willing to participate and meeting the inclusion criteria of being insufficiently physically active and perceiving no restrictions for physical activity.

| Worksite | Employees | Respondents | Respondents willing to participate | Respondents willing to participate and meeting the inclusion criteria |
| --- | --- | --- | --- | --- |
| 1 | 63 | 20 | 14 | 6 |
| 2 | 125 | 90 | 58 | 30 |
| 3 | 200 | 84 | 46 | 17 |
| 4 | 142 | 57 | 41 | 18 |
| 5 | 127 | 52 | 30 | 10 |
| 6 | 144 | 69 | 43 | 18 |
| 7 | 50 | 26 | 22 | 4 |
| 8 | 40 | 22 | 18 | 4 |
| 9 | 400 | 150 | 84 | 27 |
| 10 | 32 | 21 | 13 | 7 |
| 11 | 39 | 24 | 15 | 7 |
| 12 | 180 | 64 | 51 | 17 |
| 13 | 40 | 19 | 12 | 6 |
| 14 | 40 | 20 | 17 | 12 |
| 15 | 47 | 24 | 16 | 8 |
| 16 | 66 | 33 | 27 | 5 |
| 17 | 55 | 21 | 17 | 4 |
| 18 | 50 | 17 | 13 | 6 |
| 19 | 60 | 27 | 14 | 2 |
| 20 | 330 | 152 | 95 | 33 |
| **Total** | **2230** | **992** | **646** | **241** |
